# Implementation and workflow strategies for integrating digital therapeutics for alcohol use disorders into primary care: a qualitative study

**DOI:** 10.1186/s13722-023-00387-w

**Published:** 2023-05-08

**Authors:** Jessica M. Mogk, Theresa E. Matson, Ryan M. Caldeiro, Angela M. Garza Mcwethy, Tara Beatty, Brandie C. Sevey, Clarissa W. Hsu, Joseph E. Glass

**Affiliations:** 1 Kaiser Permanente Washington Health Research Institute, 1730 Minor Ave, Ste 1600, Seattle, WA 98101 USA; 2 Kaiser Permanente Washington Mental Health & Wellness Services, Renton, WA USA

**Keywords:** Alcohol use disorders, mHealth, Digital interventions, Primary care, Implementation science

## Abstract

**Background:**

Alcohol use disorders (AUD) are prevalent and often go untreated. Patients are commonly screened for AUD in primary care, but existing treatment programs are failing to meet demand. Digital therapeutics include novel mobile app-based treatment approaches which may be cost-effective treatment options to help fill treatment gaps. The goal of this study was to identify implementation needs and workflow design considerations for integrating digital therapeutics for AUD into primary care.

**Methods:**

We conducted qualitative interviews with clinicians, care delivery leaders, and implementation staff (n = 16) in an integrated healthcare delivery system in the United States. All participants had experience implementing digital therapeutics for depression or substance use disorders in primary care. Interviews were designed to gain insights into adaptations needed to optimize existing clinical processes, workflows, and implementation strategies for use with alcohol-focused digital therapeutics. Interviews were recorded and transcribed and then analyzed using a rapid analysis process and affinity diagramming.

**Results:**

Qualitative themes were well represented across health system staff roles. Participants were enthusiastic about digital therapeutics for AUD, anticipated high patient demand for such a resource, and made suggestions for successful implementation. Key insights regarding the implementation of digital therapeutics for AUD and unhealthy alcohol use from our data include: (1) implementation strategy selection must be driven by digital therapeutic design and target population characteristics, (2) implementation strategies should seek to minimize burden on clinicians given the large numbers of patients with AUD who are likely to be interested in and eligible for digital therapeutics, and (3) digital therapeutics should be offered alongside many other treatment options to accommodate individual patients’ AUD severity and treatment goals. Participants also expressed confidence that previous implementation strategies used with other digital therapeutics such as clinician training, electronic health record supports, health coaching, and practice facilitation would be effective for the implementation of digital therapeutics for AUD.

**Conclusions:**

The implementation of digital therapeutics for AUD would benefit from careful consideration of the target population. Optimal integration requires tailoring workflows to meet anticipated patient volume and designing workflow and implementation strategies to meet the unique needs of patients with varying AUD severity.

**Supplementary Information:**

The online version contains supplementary material available at 10.1186/s13722-023-00387-w.

## Contributions to the literature

- Provides insights regarding the best ways to implement app-based treatments for alcohol use disorder (AUD) in primary care.
- Represents the perspectives of a variety of stakeholders including care delivery leaders, clinicians, and implementation staff.
- Identifies the following recommendations to improve the implementation of digital therapeutics for AUD: (1) plan for high patient volume, (2) support teams during implementation, and (3) account for individual patient needs.

**Supplementary Information:**

The online version contains supplementary material available at 10.1186/s13722-023-00387-w.

## Background

Alcohol use disorders (AUD) are prevalent and often go untreated; in 2019, 14 million US adults had AUD and only about 7.3% received any treatment in the past year [1]. Patients are commonly screened for AUD in primary care, and many are open to primary care-based treatment [2]. However, existing primary care-based treatment programs do not have sufficient capacity to meet demand [3, 4].

Digital health includes software that assists with assessment, monitoring, or treatment of health conditions [5]. Digital health may be used in conjunction with other interventions (e.g., counselling) or as a stand-alone intervention [6]. Health systems are increasingly utilizing digital health to improve treatment access, reduce costs, and provide additional options to patients [7, 8]. Digital therapeutics are a specific form of digital health that delivers evidence-based treatments aimed to treat, manage, or prevent disorders [5, 9, 10]. They are often delivered via websites or smartphone apps. Examples of digital therapeutics for AUD include kiosk-delivered brief interventions and psychosocial interventions that are packaged as smartphone apps for the purpose of treatment or ongoing support for AUD [11–14]. While research has been conducted on the effectiveness of digital therapeutics for AUD, there is limited evidence on the best strategies for their implementation [15]. Without thoughtful implementation, digital therapeutics are unlikely to be widely utilized by clinicians. The focus of this article is digital therapeutics that run on smartphone apps, henceforth “app-based treatments.”

Qualitative and other descriptive research has been used to study the implementation of digital therapeutics in primary care. Prior studies have identified barriers and facilitators to implementation, implementation strategies, delivery approaches, and workflow considerations, resulting in rich information to inform future implementation efforts [16–21]. Overall, these studies demonstrate clinician and patient willingness to try digital therapeutics, and the importance of integrating these novel treatments into existing clinician workflows. For example, Graham and colleagues identified a sub-optimal referral process in the electronic health record as a barrier to connecting patients to a digital mental health support [17]. Mares and colleagues identified physician workload as a barrier to implementing an app-based treatment for substance use disorder (SUD), and suggested behavioral health care providers may be better suited to deliver digital therapeutics [19]. In a qualitative user-centered design study, Glass and colleagues found that patients preferred clinicians offer them app-based treatments for drug use disorders during their existing primary care visits whenever possible, as opposed to learning about apps via direct-to-consumer methods (e.g., phone calls, flyers) [16]. While such findings could help inform the implementation of a digital therapeutics for AUD, it is known that implementation strategies and clinical workflows must be tailored to the populations and practice settings in which interventions take place, especially in the case of technology-based interventions which have been traditionally difficult to implement [21–24].

The goal of this study was to identify implementation needs and strategy design considerations for integrating digital therapeutics for AUD into primary care. This study used qualitative methods to elicit lessons learned from clinicians and health system leaders who had engaged in previous digital therapeutic implementation efforts. Specifically, this study followed a pilot study of the implementation of a digital therapeutic for SUD in primary care, as well as the implementation of apps for depression and anxiety, and used these recent implementation efforts as a reference point to identify (1) barriers and facilitators to implementation and (2) adaptations that would be needed to implement a digital therapeutic for AUD.

## Methods

### Setting and context

This study was conducted at Kaiser Permanente Washington (KPWA), an integrated health system with approximately 700,000 members and 30 primary care clinics. In this system, care for AUD included brief alcohol interventions, referral to integrated mental health specialists, medication, and specialist addiction treatment by referral [25]. All primary care clinics had previous experience implementing digital interventions to support mental health. This included a cognitive-behavioral treatment app for depression and anxiety called Thrive that was offered and facilitated by primary care-based licensed independent clinical social workers (LICSWs) who served as integrated mental health specialists [26, 27], and two apps that patients could download on their own via the health plan’s patient portal website: Calm, a mindfulness and meditation app [28] and myStrength^®^, an app that provides support for improving health behaviors and addressing a variety of challenges such as stress, sleep, depression, and anxiety [8, 29]. Two clinics had recently engaged in a quality improvement pilot to implement prescription digital therapeutics, reSET^®^ and reSET-O^®^, which are for SUD and opioid use disorder (OUD), respectively [30]. Among other features, these SUD apps include cognitive-behavioral skills-based content, quizzes to reinforce concept mastery, and contingency management via electronic gift cards to reward successful progression through one’s treatment plan.

The pilot implementation of reSET and reSET-O in KPWA involved a partnership between care delivery leaders and researchers, using an implementation strategy that care delivery leaders had previously used for the Thrive depression app [31]. This implementation strategy involved live clinician training and a recorded video, workflow specifications, written job aids that describe the new steps required to offer apps within existing clinical workflows, electronic health record documentation templates, and a monthly report of app use by clinicians and patients. Additional strategies were developed by researchers in partnership with care delivery leaders, including implementation support in the form of practice facilitation [32], a dedicated health coach to support patients and encourage engagement with the app [33], and audit and feedback reports [34]. LICSWs determined eligibility and offered the app to patients when clinically appropriate. Similar to the way that the health system implemented Thrive, if patients expressed interest in using reSET or reSET-O, LICSWs helped the patient set up the digital therapeutic and facilitated follow-up care to discuss clinical issues and engagement with the app. Because reSET and reSET-O require a prescription, as an additional step LICSWs entered an electronic order which routed a prescription to a clinician with expertise in SUD treatment for approval [35, 36].

### Design

This study used content analysis and a pragmatic constructivist approach to understand clinical stakeholder perspectives on how best to integrate digital therapeutics for AUD into the primary care setting [37]. Study participants were invited to reflect on their involvement and experience implementing digital therapeutics for depression, anxiety, and SUD to share lessons learned and to make recommendations for implementing a digital intervention specifically for AUD. The KPWA Institutional Review Board approved all study activities. A completed Standards for Reporting Qualitative Research checklist is available in Additional file 1.

### Sample

A purposive sample of interview participants was identified based on their involvement with previous digital intervention implementation efforts and AUD treatment expertise. Priority was given to clinicians and health system leaders who had been involved in the recent implementation of the SUD digital therapeutics. We asked interview participants for the names of other KPWA employees with relevant expertise and, from those, recruited 5 additional participants. Participants were recruited between July and November 2021.

A clinical leader (AGM) initiated recruitment with an introductory email to potential participants. Then a study team member followed up with a detailed interview invitation. Non-responders to this message received 2–3 reminder emails. Those who responded with interest were scheduled for a 30-min interview outside of their working hours.

### Semi-structured interviews

In semi-structured interviews, participants were asked to share successes and challenges from previous implementation efforts for app-based treatments and to reflect on how those learnings could be applied to the implementation of a digital therapeutic for AUD. Particular attention was paid to the recent implementation of the SUD digital therapeutics and adaptations needed for successful implementation of a digital intervention for AUD. Questions regarding adaptations were informed by the Framework for Reporting Adaptations and Modifications to Evidence-based Implementation Strategies [38]. The full interview guide is available in Additional file 2. Interviews were conducted virtually and were audio-recorded for transcription. Interviews lasted about 22 min on average. Interviewers (JM, TM) were masters-level health services researchers trained in qualitative methods and implementation science. One interviewer (JM) was also involved in the implementation of reSET and reSET-O and had worked with several of the participants in her role as a practice facilitator. To avoid biasing participants’ responses, participants whom JM had worked with were interviewed by TM. Participants received a $60 gift card for completing the interview.

During regular study team meetings, interviewers discussed themes and concepts that were arising during interviews and the need to continue recruitment. In these discussions interviewers identified concepts that had been expressed by multiple participants. Because we expected some implementation concepts to be more relevant to participants who had specific roles in the health system, we strategically shifted recruitment efforts based on these discussions to focus on balancing representation from different roles (e.g., care delivery leader vs. clinician). Meanwhile, researchers began the analysis process and confirmed themes were repeating across participants and roles. We determined data saturation had been reached when there was group consensus that many themes were recurring, and an insufficient number of new themes were being generated by new interviews to continue recruitment despite having additional eligible participants in the sample pool. [39] Recruitment stopped when researchers had completed 16 interviews.

### Data analysis

Transcripts were analyzed by JM, TM, and JG using a rapid group analysis process inspired by prior literature [40] and further refined by a qualitative methodologist (CH). A traditional qualitative analysis process typically involves iterative code development, detailed coding of transcripts, and the creation of coding summaries and analytic memos [41]. This rapid group analysis process involved five steps: (1) the development of a form to capture paraphrased themes and associated quotes from individual transcripts, (2) use of this form to synthesize data, (3) a group analysis process through which researchers grouped related themes from individual transcripts into themes that occurred across transcripts, (4) pulling together themes from the group process and themes and quotes from individual transcripts, and (5) identification and summary of the most salient, or “key” themes.

To complete step #1 above, researchers created a form in Microsoft Excel organized in a similar manner to the interview guide to capture themes and associated quotes from transcripts. A separate copy of the form was used for each transcript. Prior to coding all the transcripts, researchers piloted the form by having JM, TM, and JG all code the same 2 transcripts. This led to some reorganization of the form and the addition of a few clarifications to facilitate consistency in using the form.

The remaining transcripts were divided between JM and TM for synthesis during step #2. To increase coding rigor, for each transcript, JM or TM would fill out the form, then the completed form was reviewed by a second researcher (JM, TM, or JG) along with the transcript to make sure no themes were missed. Discrepancies were discussed and resolved in meetings.

For the group analysis step #3, themes were pulled from the forms into a virtual board organized in the same way as the analysis form. Themes appeared on the board as sticky notes that could be moved around, labelled, and grouped. Sticky notes were color-coded by participant role to visualize potential differences in themes across roles. JM, TM, and JG held a series of video conference meetings to group like themes on the virtual board as a means of identifying emergent themes and themes that occurred across transcripts. This process served as a virtual affinity diagramming process [42].

To accomplish #4 above, researchers collated documentation from the group and individual analysis processes to create a sortable Microsoft Excel sheet which included the themes that occurred across transcripts, themes from individual transcripts, and associated quotes. This sheet served a function similar to a traditional coding memo [43].

Finally, to complete step #5, researchers used the virtual board from step #3 and supporting quotes from the Excel sheet created in step #4 to identify the most salient themes. After these “key” themes were identified, as a form of member checking, the lead author (JM) presented themes to the SUD digital intervention implementation team, including three interview participants, to validate whether themes accurately represented their views and experiences [44].

## Results

Out of 28 invited participants, 16 completed interviews (57% response rate). Participant characteristics are provided in Table 1. To maintain participant anonymity, quotes are attributed to care delivery leaders (care delivery leaders and LICSW managers), LICSWs, primary care providers (PCPs), and implementation team members (medical assistants [MAs] and practice facilitator). Additional quotes are provided in Table 2. Nine participants were directly involved in the implementation of reSET and reSET-O including two of the care delivery leaders, three LICSWs, one PCP and all three of the implementation team members. All participants besides the implementation team members had been a part of the implementation of Thrive and the apps available via the health plan’s patient portal website (Calm and myStrength). All but one of the LICSWs and PCPs were from two KPWA medical centers in Seattle, Washington. Care delivery leaders and implementation team members worked across KPWA.

**Table 1:** Participant characteristics

Quote attribution group	Number of participants	Group details
Care delivery leaders	5	3 care delivery leaders 2 LICSW managers
LICSWs	4	LICSWs who are integrated mental health specialists on the primary care teams
PCPs	4	PCPs included: 3 medical doctors 1 advanced registered nurse practitioner
Implementation team members	3	2 MAs (1 in a dedicated health coaching role who facilitated patient engagement with SUD apps) 1 practice facilitator

*LICSW*  licensed independent clinical social worker. *PCP*  primary care provider. *MA*  medical assistant. *SUD* substance use disorder

**Table 2:** Interview themes with exemplar quotes

Theme	Exemplar quote(s)
General support for implementing digital therapeutics	
Supportive of offering digital therapeutics	A lot of patients with substance use disorder or disorders show up, whether it’s alcohol use disorder or opioid use disorder or any other sorts of substances, they’re often showing up in primary care, they’re showing up in urgent care, and saying “I don’t know what to do, somebody help me.” … And so I think just having an extra tool in the toolbox to be able to refer patients back to and say that they can use this before they check into an urgent care or try to get seen, that they encourage them to start with that is also a great thing. (#11, care delivery leader) Just that we recognize that we will not be able to meet the demand for services through individual clinicians, and that we need more tools really to scale treatment and that digital tools are a great way to do that. And I think it provides a way to keep people engaged outside of sessions and to further accelerate treatment. (#13, care delivery leader) We use apps, frankly, for a lot of other diagnoses here as well—anxiety/depression, which oftentimes go along with substance use disorders. So I think [a digital therapeutic for SUD is] a very nice complement. (#9, care delivery leader)
General implementation strategy and workflow recommendations	
Partnerships with dedicated teams, champions, and researchers aid implementation	I think at the clinic what has worked well for me has been frequent meetings and support of the reSET and reSET-O researchers and the health coach. We have monthly meetings, and it's been really helpful for me to have that place to check in and have somebody outside of me tracking progress towards goals that we set or tweaking the rollout, what’s working, what's not, how do we make this easier and more accessible. (#7, LICSW) Having a point person who’s kind of the champion and can answer my questions, that's helpful for me. (#3, PCP) I think having time as a team to set aside and not have patient care and have like most of the team members there to kind of go through what this might look like, to experiment and roll something out, and then having follow up as a team where people can ask questions. (#2, PCP)
Increase knowledge about new digital therapeutics among all primary care team members	Clinicians notoriously need multiple mediums to get everyone on the same page, so it’s a combination of large group announcements, huddle cards*, coming to the morning huddles and telling people and clinicians about this. (#10, PCP) I think perhaps having a huddle card, letting all staff know what is the reSET and reSET-O program, who do we offer it to, what it entails, how it can be helpful to the patients, which providers are part of it or who do we forward this information to once patients are interested? Maybe like a huddle card is going to be the best way to go about it, so that we have some reading material that we can always go back to and read if we need a little bit refresher, so that everybody is aware that something like this is available and we can offer. (#8, MA) *Huddle cards are documents that contain a concise written overview of treatments or clinic updates on a single page. Huddle cards are often presented at short team meetings called huddles
Optimize workflows and access to information	Providers would need to have some really good training on the use of the app and then how to get people signed up easily, because when people can’t sign up easily, they just get frustrated. It needs to be user friendly. (#5, care delivery leader) And then it’s very helpful to be able to have information that’s easily accessible to share with patients, as well as for me to be able to refer back to easily. (#3, PCP) Actually we’ve set up a good process to be able to offer this to patients… Our internal standing order [in the electronic health record], for instance, to allow social work to be able to offer this to patients, I think that has gone well. (#12, care delivery leader)
App design and target population will determine implementation needs	
The app’s target population will determine implementation needs	I think a key question is if the app is designed and targeted for people who have alcohol use disorder versus just unhealthy alcohol use. If it's just unhealthy alcohol use, that’s a huge population and there would need to be something that is completely self-directed and available on the Web, and we could suggest that people go there, because that volume of patients we couldn’t manage anywhere near the way that we do people who have alcohol use disorder. [But] the population with a use disorder would benefit from having some staff who are supporting people in using the app and connecting to other care providers and supports if they are identifying a need and a desire for that. Because again, you're talking about a group of people who have a use disorder with a lot of morbidity associated with it and even a highly effective app is not likely to be effective in and of itself for most people. (#12, care delivery leader) I could see [an app offered for unhealthy alcohol use and AUD] being more available to more patients, but then you’d also need the staffing to support that if more and more patients were interested or providers were keeping it in mind more often, to recommend. (#7, LICSW)
Desire for flexibility in who to offer apps to (as opposed to strict eligibility requirements)	Frankly, in my opinion, if [the app is] tried and true … for one kind of an addiction, why not the next? (#9, care delivery leader) There's definitely been situations where I'm glancing at a chart and I’m like oh, this person would be perfect for this, for reSET, and then I go in to check what substances they're using, and it's just alcohol. So there’s a lot of people who aren’t getting enrolled, who I think would benefit from it. (#6, LICSW) I tend to think of a lot of these apps and these different things that we offer to patients as things to try for coping. It's hard to see the downside of at least trying it out [using reSET for patients with AUD only]. It seems like a lot of what reSET is aiming for is in line with what we would also offer to somebody who is using alcohol in an unhealthy way or wanted to reduce their alcohol use. (#7, LICSW)
Messaging about the app should make it clear which patients are best suited	I think really having targeted information about which patients this would be useful for, what's the evidence behind it. (#10, PCP) Since we’re offering [multiple apps for different conditions], we have to have really good knowledge about each one of those apps and what they do. (#8, MA)
Implementation adaptations for app-based AUD treatment may not need to be extensive	
Implementation of a digital therapeutic for AUD could be similar to implementation of a digital therapeutic for SUD	I think it would be a pretty similar workflow because again, there’s kind of the screener tools that typically the MA’s are using when a patient comes in for a visit. The PCP might talk to the patient and kind of introduce the idea of a digital therapeutic and then be referred or have some sort of handoff to a social worker. So I think it would be a very similar process, and I think a lot of what we're learning in the implementation of reSET and reSET-O could then be used as a foundation for implementing a new therapeutic for unhealthy alcohol use. (#15, implementation team member) Interviewer: Earlier you mentioned things like training, huddle cards, emails or announcements from partners in the delivery system. Are there any things related to those types of implementation strategies that might need to be changed? Participant: No, I think those work pretty well. (#13, care delivery leader) I’m also thinking in terms of follow up care. I think it would look the same—if I’m checking in with a patient with alcohol use disorder, it’s going to be a pretty similar follow up to substance use disorder. (#1, LICSW)
Implementation adaptations for app-based AUD treatment to accommodate high patient volume	
Simplify the workflow to accommodate higher volume	We’re already limited in how many people we can offer this to for other substance use, and if we look at people that have primary alcohol use disorder or alcohol use disorder alone, that population sort of dwarfs the overall other drug use disorder patient population who we currently can offer reSET and reSET-O to. So we would be even more challenged. Adaptation-wise, we probably would want to think about changing this a little bit so that it's even more easily administered to link people up. I kind of wonder about a virtual-only way of connecting patients with this. (#12, care delivery leader) I do think that alcohol use disorder is a lot more common than drug use disorder so there's potential for our system to be somewhat overwhelmed if we're getting lots and lots of information from patients who are in our panels who are using this and have alcohol use disorder. There's a potential for there to be basically information overload from that. (#4, PCP)
Have a dedicated, centralized staff member to manage app-based care	Ideally, we would be able to offer this with a completely remote or virtual implementation, or offering to connect the patient. Something like a centralized provider team that is able to offer patients the product, connect to it, and if it's useful, some ongoing monitoring with them. (#12, care delivery leader) I think what would be the most helpful is if there were one LICSW who is going to do this for multiple clinics and actually dedicate their time to that, it would be a way more successful program. Right now there’s just way too many competing needs. (#6, LICSW)
Provide opportunities for patients to access the app without going through a clinician	Most patients are certainly familiar with being able to go on a smart phone and download an app and figure that piece out, so if that's something that is easily doable-I understand that might be necessary in terms of making it available for free, but the more that we can make it available to them, very easily accessible and without a lot of hoops to jump through. (#3, PCP)
Implementation adaptations for app-based AUD treatment to accommodate variation in AUD severity, motivation to change, and treatment goals	
Different approaches are needed for patients with different AUD severity	I think that for low-risk patients who I don’t have a serious concern for withdrawal that may need medical intervention, [app-based treatment] could be an intriguing option. Again, I think it depends on the patient, if they’re engaged, who this might work best for. (#10, PCP)
Apps should allow for tailored goal setting (not just abstinence)	I’d imagine you'd want to be able to offer that option [an app for patients who want to reduce but not stop drinking], right? And maybe different apps with different modules or guidance. Because I do think there's a number of patients where they're not ready to abstain completely, but it’s kind of a risk reduction module so any reduction would be helpful. So I can see where there might be utility to do both. (#10, PCP) So I think from the clinic point of view it would be doing what we do now, goal setting, specific goals, and talking about motivation and barriers to reach those goals and lining that up with how they’re using the app, like applying the concepts that they’re learning in the app to actually work toward those goals. (#1, LICSW)
Supportive of offering multiple apps for patients with AUD if there is good evidence to do so and it’s clear when to use which app	I personally like [having multiple digital therapeutic options] because then I’d be able to say “here are three things I can offer you that could potentially meet your needs.” And then either based on my description, they can choose, or they can go in and see which one they prefer…I just feel there isn’t one that’s the perfect thing so being able to have more than one to choose from and go “yeah, out of all of them. I think that’s the one that works for me the best.” (#16, LICSW) I don’t see a conflict [with offering multiple apps]. I just think that you would really need to make it clear like who goes where. If you have too many apps doing the same exact thing, like everyone with alcohol use has three different options, I think that’s probably going to be confusing both for patients, just because I think that choosing something once you're at that point probably feels really overwhelming to begin with…. Otherwise you're probably going to have someone that you just pick your favorite app and everyone goes to the favorite app, right? (#2, PCP)

*PCP* primary care provider, *LICSW* licensed independent social worker

Insights from interviews were grouped into six key themes (presented from more general to more specific): (1) general support for implementing digital therapeutics (n = 16), (2) general implementation strategy and workflow recommendations (n = 16), (3) app design and target population will determine implementation needs (n = 14), (4) implementation adaptations for app-based AUD treatment may not need to be extensive (n = 12), (5) implementation adaptations for app-based AUD treatment to accommodate high patient volume (n = 10) and (6) implementation adaptations for app-based AUD treatment to accommodate variation in AUD severity, motivation to change, and treatment goals (n = 10). Key themes were well represented across provider types (i.e., color-coded sticky notes on the virtual affinity diagramming board did not reveal any patterns between roles, and all of the themes included content from multiple provider types).

### General support for implementing digital therapeutics

Participants were supportive of offering digital therapeutics for SUD generally and AUD specifically, describing it as “an extra tool in the toolbox” (#11, care delivery leader) and a way to meet high treatment demand. Participants also said that digital therapeutics were a good fit for this context because clinicians in the health system were already using apps to treat anxiety and depression.

### General implementation strategy and workflow recommendations

Reflecting on the recent pilot implementation of a SUD digital therapeutic, 5 participants said that the partnership between care delivery leaders and researchers was a successful strategy because researchers evaluated the evidence-base of the apps and research funding brought in additional resources like practice facilitation, health coaching, and electronic health record programming. Participants also recommended involving clinical leaders and clinical champions in implementation efforts and making sure those responsible for implementation had dedicated time to address clinician questions and problem-solve around implementation barriers.

Participants gave advice on approaches for sharing information about newly implemented digital therapeutics and increasing clinician knowledge about them. Participants advocated for training to describe the evidence-base for the app and information about who is most likely to benefit. They also suggested that managers provide dedicated time for clinicians to test and become familiar with the app. Participants advised clinician-facing information about digital therapeutics should come in multiple forms including email “blasts” (#1, LICSW and #9, care delivery leader), documents that contain a concise written overview of the treatment on a single page, announcements in meetings, and information from clinical champions. Participants also suggested clinicians be given an “elevator pitch” (#7, LICSW), meaning a concise verbal description of the app, that they can share with patients.

To ease implementation, participants suggested clear, simple workflows and electronic health record supports to make it easy to connect patients to the app. For example, during Thrive and reSET and reSET-O implementations, a programmer created auto-populating text about the apps for clinical notes; this made it easy for clinicians to share information about the apps and for patients and clinicians to access the information in the future.

### App design and target population will determine implementation needs

Participants explained how an app’s design, and specifically, its target population, would determine the number and characteristics of patients who might be offered the app and the supports they may need, which in turn would determine the ideal implementation strategies. For example, a digital therapeutic might be designed for patients with unhealthy alcohol use (likely a large population) or eligibility may be limited to patients with a clinically recognized AUD diagnosis (likely a much smaller population); successful implementation for these unique target populations would likely require different implementation strategies and supports for app delivery.

In general, participants preferred flexible and inclusive eligibility criteria for digital therapeutics. Participants expressed feeling challenged when digital therapeutics had eligibility criteria that restricted its use to a specific population of patients which they perceived as unnecessarily narrow. One participant remarked,

*One of the challenges we've come across so far… is that when patients present and they have alcohol use disorder, it has to be paired with another substance [for them to be eligible for reSET]. Which is really hard, because there are so many patients who have presented that have problems with alcohol use, they want support around it, and then we review and see—oh wait, they don't have another substance they're using so I can't offer them reSET.* (#11, care delivery leader)

Although reSET was designed to treat SUD but not AUD alone, participants articulated a preference to make their own determination about who to prescribe the app to. Nine participants said they thought the principles behind treating AUD and other SUDs were similar enough that it would be appropriate to prescribe reSET to patients with AUD who do not use other drugs, even though the app is not indicated for patients who solely use alcohol [30].

Whatever the eligibility criteria for an app-based treatment may be, participants stressed that messaging during implementation should make it clear to clinicians which patients are eligible and best suited for the app, especially if care teams have access to multiple digital therapeutics.

### Implementation adaptations for app-based AUD treatment may not need to be extensive

Most participants (12/16) said that few, if any, modifications would be necessary to use the existing implementation and workflow strategies developed for prior implementations of other apps to implement a digital therapeutic for AUD. Specifically, implementation strategies (e.g., clinician training materials, electronic health record note templates), and workflows for identifying patients and connecting them to the digital therapeutic (e.g., PCP identification of potentially eligible patients and referral to an LICSW) were identified as applicable for the implementation of digital therapeutics for AUD. Participants also felt that procedures for treatment and follow-up needed few modifications to implement a digital therapeutic for AUD as opposed to SUD. For instance, participants thought that an app could be used as an adjunct to usual treatment for AUD, like it had been for SUD.

Though participants generally endorsed the applicability of past implementation strategies, many also recommended changes to account for the large number of patients with AUD and the unique treatment needs of patients with varying AUD severity, motivation to change, and treatment goals. These recommendations are described in detail below.

### Implementation adaptations for app-based AUD treatment to accommodate high patient volume

Most participants (10/16) expected more patients would be eligible for and interested in app-based treatment for AUD compared to SUD because AUD is more prevalent. Participants recommended adaptations to help care teams manage higher patient volume. To improve a health system’s capacity to offer the app to more patients, several participants advocated for a ‘no wrong doors’ approach where any care team member could connect the patient to the app. One participant shared,

*We talk about clients having rapport with their PCP... that's not always the case. It may be the nurse or the social worker or the therapist who has far more contact with the client… I believe this is something all clinicians should have awareness of in their toolbox, so to speak, so that if they have rapport with their particular client, that they feel comfortable discussing and offering it.* (#9, care delivery leader)

On the other hand, others thought that it would be easier to manage high patient volume if there was one dedicated person (e.g., a centralized LICSW or MA) who would be responsible for connecting patients to the app for multiple clinics. This would help ensure that patients could be reached even if clinicians in the local clinic were too busy to offer the app.

Some participants suggested patients should be able to access the app without going through a clinician and had specific ideas for how a digital therapeutic could be paired with existing wellness or treatment resources. For example, patients at the study site can complete an annual health profile online that includes an alcohol screening instrument. When patients are screened for unhealthy alcohol use, they could be offered the app algorithmically based on a positive screening score result. Another participant suggested,

*If somebody is prescribed a medication to reduce cravings, [we could] also hand them this brochure [with information about an app]… at minimum, give them this brochure, at maximum have a quick conversation about here's something else we could pair with medication. (#7, LICSW).*

The potential for a high volume of patients caused participants to express doubt care teams would have the capacity to actively monitor patient app use. To support care teams in working with patients using digital therapeutics, participants recommended giving clinicians additional dedicated time to care for these patients, including time to view and process information the app collects (if applicable). Participants also suggested adding supports for patients who are engaging in the app such as tech support or access to a health coach who could monitor patient app use.

### Implementation adaptations for app-based AUD treatment to accommodate variation in AUD severity, motivation to change, and treatment goals

Interview participants recommended digital therapeutics be offered as one of many options for AUD treatment, depending on the individual patients’ needs. Different treatment options are needed to account for individual patients’ AUD severity, motivation to change, and treatment goals in terms of whether they want to stop versus reduce their drinking. One participant remarked,

*I think a key question is if the app is designed and targeted for people who have alcohol use disorder versus just unhealthy alcohol use. If it's just unhealthy alcohol use, that's a huge population and there would need to be something that is completely self-directed and available on the Web…. [But] the population with a use disorder would benefit from having some staff who are supporting people in using the app and connecting to other care providers and supports if they are identifying a need and a desire for that. Because again, you're talking about a group of people who have a use disorder with a lot of morbidity associated with it and even a highly effective app is not likely to be effective in and of itself for most people.* (#12, care delivery leader)

Two participants hypothesized that app-based treatments would be best for patients with mild to moderate AUD who do not need formal treatment. One LICSW shared,

*There's a lot of people who get in touch with their provider, their provider gets in touch with social worker because they've started to have the conversation around 'maybe I'm drinking a little bit too much, but I'm not drinking so much that I need treatment or that I need to be connected to a substance use therapist, but maybe I just need a little bit of something to help me get back on track with my goals around a healthy relationship with alcohol.' And so, I think those are patients who would be particularly receptive to app-based care. Because to them it doesn't feel like it's a major problem. It's like the level of treatment fits the level of problem.* (#6, LICSW)

On the other end of the spectrum, participants were skeptical about the effectiveness of the app for patients with more severe AUD and expressed concerns about what would happen in a crisis. Some conveyed that dangerous withdrawal symptoms are of greater concern with AUD than SUD, and participants stressed that patients at risk of severe withdrawal symptoms should not rely on digital therapeutics alone for AUD treatment. One PCP said, “I'd probably just want to screen for more of those medically concerning signs of withdrawal, so then they could be encouraged to seek medical care if they are happening…” (#10, PCP).

Participants also hypothesized that patient motivation to change is a determining factor in whether a digital therapeutic would be effective. In general, participants did not think app-based treatment would be useful to patients with low motivation to change. For example, one social work manager shared, “I think that the people who are heavily drinking… I think the app might not be powerful enough” (#5, care delivery leader). Another LICSW compared patients with low motivation to change to patients with high motivation to change:

*I think there are some patients that don't want to do anything about their drinking. They either don't acknowledge that it's a problem, or they acknowledge it’s a problem, but they don't want to do anything about it. There are other patients that are like 'yeah, I recognize that I have a problem, but I don't want to go to inpatient, I don't want to go to a facility, I don't want to do all of that, I want to be able to do it on my own.' So those patients I think would be appropriate. Yeah, that's great - you have that motivation, you're driven, you want to make a difference, but you want to be able to have it be a little less intensive. I think a resource like that could be really beneficial.* (#16, LICSW)

Participants were supportive of offering app-based treatments to patients who wanted to reduce but not stop their drinking, and a few participants emphasized the importance of allowing for goals besides abstinence. One care delivery leader shared,

*You would want a tool that would allow patients to have different goals in terms of what they're looking at with their alcohol use, both from a patient-centered standpoint but also from an effectiveness standpoint, because again we know that just helping people significantly reduce their alcohol use has tremendous health benefits for people.* (#12, care delivery leader)

Participants were supportive of having multiple apps on hand to offer to patients with AUD (6/16), as long as it is clear to clinicians “who goes where” (#2, PCP), or which patients to connect to which apps.

## Discussion

This study used qualitative methods to elicit perspectives on the implementation of a digital therapeutic for AUD from care delivery leaders, clinicians, and implementation staff who had experience implementing other app-based treatments, including a digital therapeutic for SUD. Overall, participants felt that the strategies and general workflow procedures for implementing digital therapeutics for AUD could be similar to those used for digital therapeutics for SUD. However, participants articulated that the amount of support needed to promote a successful implementation could be much higher for AUD, and also identified important population characteristics (e.g., patient addiction severity) that must be considered when implementing apps to treat AUD.

### High demand for digital therapeutics for AUD may necessitate particular implementation supports

One special consideration for implementing a digital therapeutic for AUD identified in this study is the need to accommodate a high volume of patients. AUD is indeed more prevalent than SUD in the United States overall [45] and in primary care. One multisite study estimated that 13.9% of primary care patients had past-year AUD, which was approximately double that of other individual drug use disorders (e.g., 7.4% cannabis, 5.1% cocaine, 3.3% heroin, and 2.4% prescription opioids) [46]. Participants offered a few different ideas to accommodate high patient volume including (1) training all care team members to be able to offer digital therapeutics to avoid workflow bottlenecks, (2) implementing a digital therapeutic through a dedicated, centralized clinician to avoid bandwidth issues in local clinics, (3) providing opportunities for patients to access the app without going through a clinician, and (4) providing dedicated time for local teams or a dedicated centralized health coach to handle tasks such as helping patients with technical problems and monitoring app use. Increasing staffing of mental health specialists within primary care may be the most supportive way to meet the needs of large numbers of patients, but this may be difficult to accomplish in the near-term given the challenges recruiting for and retaining qualified workers in demanding and generally low-paying behavioral health positions [47–49].

The idea that local primary teams may have difficulty offering apps to patients and guiding them through app use is supported by the literature. For example, Graham and colleagues had planned to recruit patients into a digital therapeutic study from primary care by having clinicians recommend the study and the app and placing an order in the electronic health record. However, only 5% of referrals during the study came through this mechanism. Instead, direct-to-consumer techniques (e.g., digital and print media, registry emails) had the highest yield [17]. Other studies have reported needing to change implementation plans due to workload concerns within clinics. For instance, Mares and colleagues reported their implementation plan of a digital therapeutic in Federally Qualified Health Centers was to involve PCPs, but in the end mental health specialists took on the work of connecting patients to app-based treatments [19]. On the other hand, a qualitative study that elicited patient preferences using user-centered design methods found that primary care patients with drug use disorders wanted their own clinician(s) to offer them apps, largely because they felt they could trust these clinicians and benefit from existing relationships with them [16]. Taken together, these findings suggest health systems implementing digital therapeutics should invest in multiple avenues for connecting patients with apps by both providing tangible support to clinicians who are expected to incorporate apps into patient care (such as dedicated time) and developing other pathways for patients to access digital therapeutics (such as a centralized staff member or direct to consumer techniques).

### Implementation strategies should consider the unique needs of patients with varying AUD severity

Findings in this study suggest that the severity of a patient’s AUD should inform decisions on whether to offer them digital therapeutics and/or what follow-up would be needed if they choose to engage in app-based treatment. Some participants expressed concern that app-based treatment would be inappropriate for patients with severe AUD. In a prior qualitative study that interviewed primary care patients with depression, participants said that apps may not be suitable for patients experiencing severe depression because they may not be motivated to engage with app-based treatment [18]. However, in another study that interviewed primary care patients about their preferences for using apps for drug use disorder in primary care, participants said that patients with severe drug use disorder could be given an app for treatment provided they were also provided with additional support and follow-up [16]. In the current study, clinicians mentioned alcohol withdrawal as one factor that would not necessarily preclude use of an app-based treatment but would necessitate additional monitoring and follow-up.

While previous qualitative studies have identified the importance of tailoring app delivery to accommodate patient motivation [16], previous studies have not examined patient motivation as it relates to AUD treatment decisions regarding digital therapeutics. Several clinician participants speculated that app-based treatments may be uniquely suited for patients with high motivation to change who may be unwilling or unable to commit to intensive forms of treatment. Inpatient treatment in particular may be viewed as time consuming and expensive, and previous research has found patients prefer flexible AUD treatment options [50]. Future research may wish to investigate the patient acceptability, effectiveness, and safety of using digital therapeutics with clinician support on an outpatient basis as an alternative to, or as a prelude to more intensive forms of treatment.

Participants also recommended direct-to-consumer techniques to connect patients with unhealthy alcohol use to app-based treatments. This approach is being trialed in New Mexico State. Starting in 2020, the New Mexico Human Services Department launched the 5-Actions Program™ which provides a digital and phone-based support for people seeking care for unhealthy alcohol or substance use [51]. Future research should evaluate the effectiveness of this program and other direct-to-consumer approaches for offering app-based treatments for unhealthy alcohol use in different settings (e.g., state vs. healthcare sponsored).

## Limitations

This study has limitations. Participants were recruited from a single regional integrated health care system in Washington state. Findings may not be generalizable to other geographical areas and types of health care systems. While it is a strength that this study included a diversity of roles (e.g., care delivery leaders, PCPs, and LICSWs), there were low numbers of participants for some roles (i.e., 2 LICSW managers, 2 MAs, 1 practice facilitator). While all participants had experience working in proximity to apps for depression, anxiety, or SUD, only 8 were involved in the implementation of two prescription digital therapeutics (reSET and reSET-O) for SUD. Finally, during analysis it was sometimes difficult to differentiate between participant comments specific to the implementation of apps for AUD and general advice for implementation efforts related to apps for any health condition.

## Conclusion

If implemented appropriately, digital therapeutics could be used to provide effective treatment for AUD within primary care. Participants thought that training, electronic health record tools and templates, practice facilitation, health coaching, protected clinician time, and having dedicated clinicians to offer apps could be effective implementation strategies for apps for AUD. The approach for connecting patients to digital therapeutics for AUD must be tailored to accommodate the anticipated high patient volume while minimizing the workload burden of busy care teams. Digital therapeutics and their delivery should also be tailored to meet the needs of patients with varying AUD severity. Findings may be used to inform future efforts to implement digital interventions for AUD into primary care.

## Supplementary Information

**Additional file 1.** Standards for Reporting Qualitative Research checklist. Microsoft word document (.docx).

**Additional file 2.** Interview guide. Microsoft word document (.docx).

## Data Availability

The interview materials are provided in Additional File 2. Additional study materials are available from the last author upon reasonable request. Data used in the current study are not publicly available to protect participant privacy.

## Abbreviations

AUD: Alcohol use disorder
KPWA: Kaiser Permanente Washington
LICSW: Licensed independent clinical social worker
MA: Medical assistant
OUD: Opioid use disorder
PCP: Primary care provider
SUD: Substance use disorder
