# Supplementary material for: Implementation and workflow strategies for integrating digital therapeutics for alcohol use disorders into primary care: a qualitative study
Source: Addict Sci Clin Pract. 2023 May 8;18:27. doi: 10.1186/s13722-023-00387-w (PMC10169300; doi:10.1186/s13722-023-00387-w)
Supplement: Supplementary file 2 — Additional file 2. Interview guide. Microsoft word document (.docx). [file 13722_2023_387_MOESM2_ESM.docx]

**Implementing Digital Treatments for Alcohol Use Disorder**

**Interview Guide**

**Introduction**

Welcome. Thank you for agreeing to talk with me today.

We are interviewing people with different perspectives regarding the implementation treatment apps for patients with drug use disorders. We want to hear your perspectives to help us learn how Kaiser Permanente can better offer app-based treatments and how similar services can be used with unhealthy alcohol use.

Are you familiar with reSET and reSET-O, and how it’s being used at your clinic?

(if “yes”) Great, we will mostly be asking you to reflect on what you know from the implementation of reSET/reSET-O, but you may also think about other app-based health interventions (e.g., Thrive, myStrength, Calm).

(if “no”) Okay, no worries. For context, reSET and reSET-O are app-based treatments for substance use disorder (reSET-O is specific to opioid use disorder, and reSET is for patients with other substance use disorders). The social worker at your clinic can prescribe reSET or reSET-O to patients who are eligible and open to app-based treatments. For this interview, you can think about any app-based health intervention you are familiar with (e.g., Thrive, myStrength, Calm) or just speak from your experience with patient care more broadly.

We would like to audio record and professionally transcribe these conversations. The purpose of audio recording and transcription is to help us researchers remember the conversation accurately. We may use anonymous quotes from the transcripts when we publish our results or share with other people what we learn about implementation, but no study reports will include your name or other information that identifies you. You are also free to skip any question you don’t want to answer. Do I have your permission to audio record this conversation?

**Yes______ No______**

*(if “yes”)* Thank you. I’m going to turn off my camera so that it’s easier for us to transcribe the audio recording—if you can please do the same, I’ll start the recording now*. -- Start recording --*

*(if “no”)* Is it okay if I take notes during our call? *(if “no” again)* If we can’t record, detailed notes are really important for us to capture learnings from the interview. It sounds like participating in this is not a good fit for you right now. If you change your mind or know anyone else who may be interested in sharing their perspectives feel free to reach back out, but I think we can end the interview here.

Do you have any questions for me [us] before we start?

**“Grand Tour” Questions**

I would like to begin by asking about your perspective about the implementation of reSET and reSET-O

[IF NEEDED*:* By implementation, we mean the training, tools, workflow, delivery, and use of this app]

1. **What is your understanding of the implementation of reSET and reSET-O?**

**Prompt:** What role if any did you have in implementation?

1. **What worked well with the implementation of reSET and reSET-O? What was challenging or what didn’t work so well?**

**Prompts:**

- What did you like about the process of using/offering reSET to patients? What did you not like about the process of using/offering reSET to patients?
- What would have improved the implementation of reSET?
- How did it fit with your clinic’s approach for offering substance use treatment?
  - - Other probes: How does it fit with how KP as an organization provides care? It is a good fit for how healthcare in the US works in general?
  - Answer from your perspective (may be more high-level or on-the-ground understanding of the implementation)

Now I would like to switch our focus to alcohol use disorders:

1. **How do you feel about offering** **digital therapeutics like reSET for treatment of unhealthy alcohol use?**

[IF NEEDED*:* By digital therapeutics, we mean software such as websites or apps used by patients to improve health outcomes.]

**Prompts:**

- Would treatment with a digital therapeutic look any different for patients with unhealthy alcohol use than patients with drug use?

**Optional Follow-Up:**

- reSET has FDA clearance for treatment of unhealthy drug use, but it did not seek clearance for treatment of unhealthy alcohol use. How do you feel about off-label use of reSET for unhealthy alcohol use? [IF NEEDED: off-label means using an FDA-approved drug for an unapproved use]
- How do you think that reSET might enhance the care you offer for alcohol use disorders?

[IF NEEDED: The reason reSET has not been FDA approved for treatment of alcohol use disorder is the study was funded by the National Institute of Drug Use, so the research focused on drugs not alcohol. Although no participants in the trial had AUD only (without drug use), about one third of the sample had co-occurring AUD, often as their primary diagnosis.]

1. **What should be changed to improve implementation of a digital therapeutic for unhealthy alcohol use? By implementation, I mean things other than changing the app itself but rather how it is integrated into routine care.**

[IF NEEDED: Implementation includes things like training, huddle cards, changes to workflow, announcements in meetings, etc. Some specific implementation strategies used for reSET and reSET-O include creating a group with dedicated time to work on implementation and tasking an MA with supporting patients in using the app.]

[IF NEEDED: Things that might be changed are clinical workflow (identification of patients, introduction, set-up, and follow-up), training (standard implementation), health coaching, and practice facilitation]

- *Probe on the specific strategy/implementation components if needed, but don’t ask all participants to walk you through each*

**Prompts:**

- How would you change implementation from using reSET for substance use disorders to using it for alcohol use disorders?
- What would be different about offering it to patients who want to reduce but not stop their drinking?
- Would you make that change for all ___[clinics/patients/providers]____, or just some _____?
- What led you to recommend that change?
- What other changes would be necessary to make that happen?

Use the FRAME-IS to assess need for adaptation:

- What is adapted
- Nature of that adaptation
- Goal of the adaptation
- What level does the adaptation occur (policy, health system, clinic, provider, patient)

**Other/Misc. Questions**

1. **What else would be helpful for us to know before piloting the implementation of reSET or another ‘app’ for treatment of unhealthy alcohol use?**

**Prompts:**

- What would you think about offering multiple apps for alcohol use or drug use in one clinic?

*-- Stop recording –*
